# Effect of simultaneous vaccination with H1N1 and GAD-alum on GAD_65_-induced immune response

**DOI:** 10.1007/s00125-017-4263-x

**Published:** 2017-03-29

**Authors:** Beatriz Tavira, Mikael Cheramy, Stina Axelsson, Linda Åkerman, Johnny Ludvigsson, Rosaura Casas

**Affiliations:** Division of Pediatrics, Department of Clinical and Experimental Medicine, Faculty of Medicine and Health Sciences, Linköping University, 581 85 Linköping, Sweden

**Keywords:** Children, GAD, H1N1, Immune intervention, Type 1 diabetes, Vaccine

## Abstract

**Aims/hypothesis:**

A European Phase III trial of GAD formulated with aluminium hydroxide (GAD-alum) failed to reach its primary endpoint (preservation of stimulated C-peptide secretion from baseline to 15 months in type 1 diabetes patients), but subgroup analysis showed a clinical effect when participants from Nordic countries were excluded, raising concern as to whether the mass vaccination of the Swedish and Finnish populations with the Pandemrix influenza vaccine could have influenced the study outcomes. In the current study, we aimed to assess whether Pandemrix vaccination affects the specific immune responses induced by GAD-alum and the C-peptide response.

**Methods:**

In this secondary analysis, we analysed data acquired from the Swedish participants in the Phase III GAD-alum trial who received subcutaneous GAD-alum vaccination (two doses, *n* = 43; four doses, *n* = 46) or placebo (*n* = 48). GAD autoantibodies (GADA) and H1N1 autoantibodies, GAD_65_-induced cytokine secretion and change in fasting and stimulated C-peptide levels from baseline to 15 months were analysed with respect to the relative time between H1N1 vaccination and the first injection of GAD-alum.

**Results:**

GADA levels at 15 months were associated with the relative time between GAD-alum and Pandemrix administration in participants who received two doses of the GAD-alum vaccine (*p* = 0.015, *r* = 0.4). Both in participants treated with two doses and four doses of GAD-alum, GADA levels were higher when the relative time between vaccines was ≥210 days (*p* < 0.05). In the group that received two doses of GAD-alum, levels of several GAD_65_-induced cytokines were higher in participants who received the H1N1 vaccination and the first GAD-alum injection at least 150 days apart, and the change in fasting and stimulated C-peptide at 15 months was associated with the relative time between vaccines. Neither of these effects were observed in individuals who received four doses of GAD-alum.

**Conclusions/interpretation:**

In individuals who received two doses of GAD-alum, receiving the Pandemrix vaccine closer to the first GAD-alum injection, i.e. <150 days, seemed to affect both GAD_65_-induced immune response and C-peptide preservation.

**Trial registration:**

ClinicalTrials.gov NCT00723411.

**Electronic supplementary material:**

The online version of this article (doi:10.1007/s00125-017-4263-x) contains peer-reviewed but unedited supplementary material, which is available to authorised users.

## Introduction

Preservation of residual beta cell function in type 1 diabetes may play an important role in an individual’s quality of life, avoidance of complications and even long-term survival [1], but most clinical interventions to date in individuals with recent-onset type 1 diabetes have shown no or limited efficacy [2–8]. In a Phase II trial (NCT00435981), GAD_65_ formulated with aluminium hydroxide (GAD-alum) showed efficacy in preserving residual insulin secretion in children and adolescents with recent-onset type 1 diabetes [9, 10]. However, a subsequent Phase III trial of GAD-alum (NCT00723411) failed to reach its primary outcome [11], raising the question: why did efficacy differ from that seen in the previous Phase II study? Indeed, in the Phase III GAD-alum trial the treatment had significant efficacy in several prespecified subgroups, such as participants from non-Nordic countries, but not in Nordic participants, a group that was dominated by Swedes. Prespecified exploratory analyses, including analyses of genetic risk related to HLA genotypes, did not find any associations explaining the different outcomes between Nordic and non-Nordic participants [11].

A particular feature of the Swedish and Finnish participants in the Phase III GAD-alum trial was that a vaccination campaign against the influenza A (H1N1) virus began in both countries in October 2009, following the WHO issuing a pandemic alert for the influenza strain A/(H1N1)pdm09. As a consequence, most Swedish and Finnish participants in the study had been vaccinated with Pandemrix, a vaccine that uses the potent AS03 adjuvant containing α-tocopherol, a molecule with a powerful immunomodulatory effect [12]. The ability for AS03 to promote innate immune system activation not solely at the injection site but also in non-regional lymph nodes, making it more potent than aluminium hydroxide in terms of immune system activation, has previously been described [13]. Indeed, AS03 has been associated with narcolepsy in Sweden and Finland [14]. Together, the Swedish and Finnish participants constituted nearly half of the Phase III GAD-alum study cohort (166/334 participants), raising concerns as to whether Pandemrix vaccination was one of the factors influencing the study outcome, possibly contributing to the difference seen between the Nordic and non-Nordic populations, with participants from the latter receiving another kind of influenza vaccination [11].

Although it is well known that vaccines can interfere with each other to decrease vaccination efficacy, the possible interference of vaccinations with autoantigen treatment in type 1 diabetes is a poorly explored field. In the Phase III GAD-alum trial, treatment with any vaccine within 1 month prior to the first GAD-alum dose or planned vaccinations up to 2 months after the last GAD-alum injection were not permitted, with the exception of influenza vaccination. We have previously shown that GAD-alum has a specific immunomodulatory effect, indicated by enhanced GAD autoantibodies (GADA) and specific in vitro cytokine secretion upon GAD_65_ stimulation [15, 16]. Thus, in the current study, we aimed to assess whether vaccination with Pandemrix might have interfered with the specific immune and C-peptide response to GAD-alum treatment.

## Methods

### Participants

**Fig. 1:**
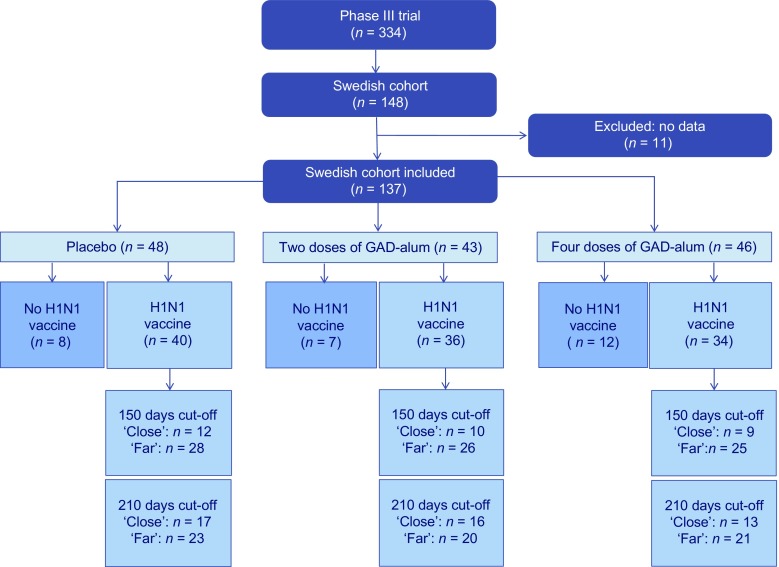
Flow chart for the Swedish participants in the Phase III trial. From the original Swedish cohort (*n* = 148), individuals included in this study (*n* = 137) were divided according to GAD-alum treatment (two doses and four doses) or placebo. Individuals within each arm were stratified according to whether they received the H1N1 vaccination or not. They were further categorised according to two different cut-off periods for time between H1N1 vaccination and the first GAD-alum injection, into ‘close’ (<150 or <210 days) or ‘far’ (≥150 or ≥210 days)

The design and characteristics of the Phase III GAD-alum trial (NCT00723411) have been previously described [11]. The study was a multicentre, randomised, double-blinded trial performed in nine European countries (Finland, France, Germany, Italy, the Netherlands, Slovenia, Spain, Sweden and the UK). Individuals (*n* = 334) aged 10–20 years with fasting C-peptide levels >0.1 nmol/l and detectable serum GADA were enrolled within 3 months of receiving a diagnosis of type 1 diabetes. Participants received either: (1) four doses of 20 μg GAD-alum on days 1, 30, 90 and 270 (the ‘four-dose’ group); (2) two doses of GAD-alum on days 1 and 30, followed by two doses of placebo on days 90 and 270 (the ‘two-dose’ group); or (3) four doses of placebo on days 1, 30, 90 and 270. The study was approved by relevant regulatory authorities and research ethics boards for the participating sites and countries. Written consent and/or assent was acquired from all participants and guardians, as required. Samples from 137 of the 148 Swedish participants in the Phase III GAD-alum trial were included in the current study (Fig. 1).

### H1N1 haemagglutinin antibodies

Influenza A/H1N1 haemagglutinin antibodies (H1N1Abs) were detected with a radiobinding assay, as described elsewhere [17]. Briefly, recombinant A/H1N1 haemagglutinin was labelled with 35S-methionine (PerkinElmer Life and Analytical Sciences, Brussels, Belgium). H1N1Abs were analysed, separating bound antibody from free antigen with protein A-Sepharose (Invitrogen, Carlsbad, CA, USA). The cut-off level for H1N1Ab positivity was 71 RU/ml [17].

### GADA, C-peptide and cytokine secretion

In this secondary analysis, GADA, C-peptide and cytokine secretion were analysed using data previously acquired for the Swedish participants in the Phase III GAD-alum trial (*n* = 137), as described elsewhere [15]. Briefly, serum GADA titres were determined using a GAD_65_ antibody ELISA (RSR, Cardiff, UK) [18]. C-peptide analysis was performed with an Immulite 2000 C-peptide kit on an Immulite 2000 analyser (Siemens Healthcare Diagnostics Product, Llanberis, UK). The clinical effect of treatment was determined via changes in stimulated C-peptide secretion measured as AUC, reported as a percentage change from baseline.

For cytokine quantification, peripheral blood mononuclear cells (PBMCs) were cultured for 7 days in the presence of 5 μg/ml recombinant human GAD_65_ (Diamyd Medical, Stockholm, Sweden) or in medium alone at 37°C in 5% CO_2_, as previously described [15, 16]. IL-1β, IL-2, IL-5, IL-10, IL-13, IL-17, TNF-α and IFN-γ were measured in cell culture supernatant fractions using a Bio-Plex Pro Cytokine Panel (Bio-Rad, Hercules, CA, USA) according to the manufacturer’s instructions. Data were collected using the Luminex 200 (Luminex xMAP; Luminex, Austin, TX, USA). The specific antigen-induced cytokine secretion level was calculated by subtracting the spontaneous secretion (i.e. secretion from PBMCs cultured in medium alone) from the one following stimulation with GAD_65_.

### Statistical analysis

As datasets were significantly different from a Gaussian distribution, as determined using the Kolmogorov–Smirnov test, non-parametric tests corrected for ties were used. Unpaired analyses for three or more groups were performed using the Kruskal–Wallis test and correlations were analysed using the Spearman’s rank correlation coefficient test. Differences between groups were calculated using the Mann–Whitney *U* test. A probability level of <0.05 was considered statistically significant. Calculations were performed using IBM SPSS Statistics version 23 (IMB SPSS, Armonk, NY, USA) and graphical representations were developed using GraphPad Prism 5 for Windows (GraphPad Software, La Jolla, CA, USA).

## Results

Samples from the Swedish participants of the GAD-alum study were stratified according to treatment arm (two doses, four doses or placebo) and further stratified by receipt of H1N1 vaccination. Participants who received the H1N1 vaccine were classified as ‘close’ and ‘far’, defined by the time between administration of the first injection of GAD-alum/placebo and H1N1 vaccination (Fig. 1).

### GADA and H1N1Ab titres and relative time between vaccinations

**Fig. 2:**
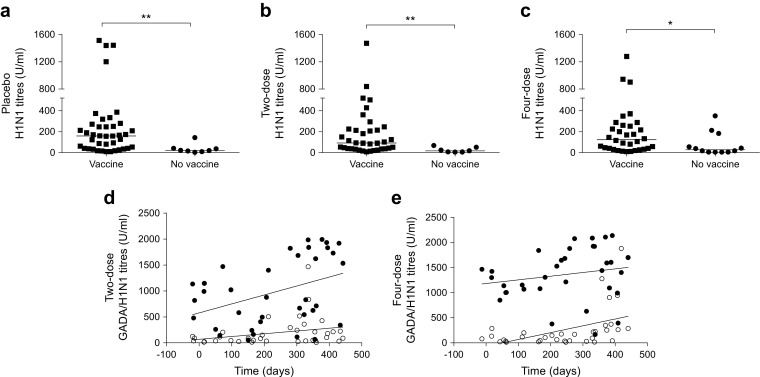
(**a**–**c**) H1N1Ab titres at 15 months in participants who received (**a**) placebo, (**b**) two doses of GAD-alum or (**c**) four doses of GAD-alum. Participants within each arm were stratified according to whether they received H1N1 vaccination or not. Median values are indicated by horizontal lines. (**d**, **e**) GADA (black circles) and H1N1Ab (white circles) titres for each participant at 15 months and their correlation with the relative time (days) between the first injection of GAD-alum and H1N1 vaccine in (**d**) the two-dose group (GADA, *p* = 0.015, *r* = 0.4; H1N1, *p* = 0.079, *r* = 0.29) and (**e**) the four-dose group (GADA, *p* = 0.16*,r* = 0.24; H1N1, *p* = 0.001, *r* = 0.53). *p < 0.05, **p < 0.01 vs not vaccinated

The analysis of H1N1Abs in serum samples collected at baseline and at 3 and 15 months of the Phase III GAD-alum trial showed, as expected, that antibodies against H1N1 increased in participants who received the influenza vaccine, with significant differences at 15 months between those who were vaccinated and those who were not (Fig. 2a–c). We have previously shown that GADA levels are induced by both two and four doses of GAD-alum, but not by placebo [15]. In the current study, the GADA titres at 15 months (the main study period) did not differ significantly between participants who did and did not receive the H1N1 vaccine in any of the groups (electronic supplementary material [ESM] Fig. 1).

H1N1Ab titres at 15 months were associated with the relative time between the first injection of GAD-alum and H1N1 administration in the four-dose group (*p* = 0.001, *r* = 0.53), but not in the two-dose group (Fig. 2d, e). The analysis of GADA levels at 15 months showed an association of GADA with the relative time between GAD-alum and H1N1 injections in the two-dose group (*p* = 0.015, *r* = 0.4; Fig. 2d). A similar relationship seemed to exist in the four-dose group, without being statistically significant (Fig. 2e).

**Fig. 3:**
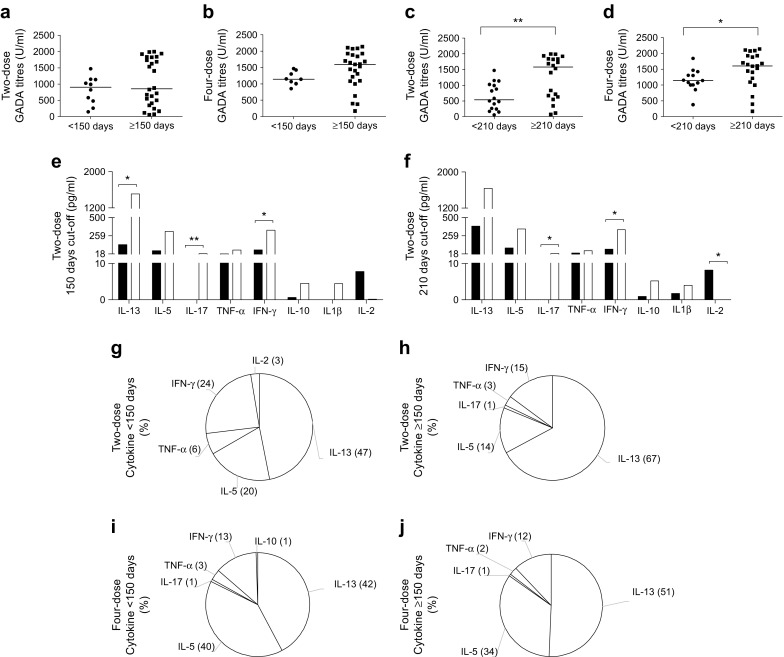
(**a**–**d**) GADA levels at 15 months in the (**a**, **c**) two-dose and (**b**, **d**) four-dose GAD-alum recipients who received the H1N1 vaccine. Participants from both groups were divided according to the relative time between influenza vaccination and the first GAD-alum injection to define ‘close’ (<150 and <210 days, circles) and ‘far’ (≥150 and ≥210 days, squares) vaccinations. (**e**, **f**) Cytokine levels in H1N1-vaccinated participants from the two-dose group according to the ‘close’ (black bars) and ‘far’ (white bars) vaccination cut-off: (**e**) 150 days and (**f**) 210 days. (**g**–**h**) Levels of antigen-induced cytokine secretion upon in vitro PBMC stimulation with GAD_65_ are given after subtraction of spontaneous secretion. IL-13, IL-5, IL-17, TNF-α, IFN-γ, IL-10, IL-1β and IL-2 were detected using Luminex. Relative contribution (%) of each cytokine to the GAD_65_-induced cytokine profile in (**g**, **h**) two-dose and (**i**, **j**) four-dose GAD-alum recipients according to the 150 days cut-off are represented by pie charts. For (**a**–**e**), data are presented as median values. *p < 0.05 and **p < 0.01, ‘close’ vs ‘far’ H1N1 vaccination

Because the exploratory analyses of the Phase III GAD-alum results showed that removing individuals who received an H1N1 vaccination within 150 days of the initial treatment tended to improve the estimate of treatment ratio (i.e. treatment effect on C-peptide preservation) [11], we used 150 days as a cut-off point to define the relative time between injections as ‘close’ (<150 days) or ‘far’ (≥150 days) from each other. The analysis of GADA levels using this definition showed that GADA did not differ between participants who received vaccinations ‘far’ from compared with ‘close’ to GAD-alum treatment in either group (Fig. 3a, b). Since relatively few participants received the two vaccinations within 150 days of each other, the use of a later cut-off point at 210 days for defining ‘close’ and ‘far’ rendered groups that were more even in number. The analysis using this cut-off revealed that GADA levels were higher in participants receiving the GAD-alum and H1N1 treatments more than 7 months apart than in those who received them within 7 months, both in the two-dose and four-dose groups (Fig. 3c, d).

### Cytokine secretion and influenza vaccination

Assessment of PBMC cytokine secretion during the Phase III GAD-alum study showed that GAD-alum treatment had a specific immunomodulatory effect, as shown by in vitro GAD_65_-induced cytokine secretion [15]. Comparison of GAD_65_-induced cytokine levels at 15 months did not reveal any difference between participants who did or did not receive the influenza vaccination (data not shown).

To determine whether the relative time between the vaccinations and GAD-alum treatment had an impact on GAD_65_-induced cytokine secretion, we compared cytokine levels between participants who received the H1N1 vaccine ‘close’ to (<150 days) and ‘far’ from (≥150 days) the first GAD-alum injection. Within the two-dose group, we observed higher levels of IL-13, IFN-γ and IL-17 in the ‘far’ vaccinated participants compared with those vaccinated ‘close’ to GAD-alum treatment (Fig. 3e). In addition, IL-5, IL-10 and IL-1β seemed to be increased in the group who received H1N1 vaccinations ‘far’ from GAD-alum treatment, although this did not reach statistical significance (Fig. 3e). Analysis of cytokine levels using the 210 days cut-off to define ‘close’ and ‘far’ vaccinations in the two-dose group revealed a similar pattern to that observed with the 150 days cut-off (Fig. 3f). In contrast to other cytokines, IL-2 was higher in the participants vaccinated ‘close’ to GAD-alum treatment using both cut-off values (although this was only significant using the 210 days cut-off). This is very interesting considering the lack of effect seen in the four-dose group, where no differences were observed between participants who received the vaccinations ‘close’ and ‘far’ from GAD-alum treatment, regardless of which cut-off point was applied (ESM Table 1).

### Cytokine profile and relative time between vaccinations

We have previously reported that the cytokine profile after GAD-alum administration tends to switch from a wide cytokine profile towards a more predominant T helper (Th)2-associated profile from baseline to 21 months [15]. Thus, we next examined the relative cytokine contribution to total GAD_65_-induced cytokine secretion in participants who received H1N1 vaccination in relation to the relative time to the first GAD-alum injection. Using the 150 days cut-off period, we observed that participants in the two-dose group who received the influenza and GAD-alum injections ‘far’ apart displayed a more pronounced Th2-associated profile than participants receiving them ‘close’ (Fig. 3g, h). No differences in the relative cytokine contribution to total GAD_65_-induced cytokine secretion were detected in the four-dose group (Fig. 3i, j).

### GADA and cytokine levels in ‘far’ vaccinated individuals

Correlation analysis of GADA levels at 15 months revealed two distinct clusters of ‘far’ vaccinated participants in the two-dose group using the 210 day cut-off, raising the question as to whether they had some common phenotypic or immunological features. A cut-off at 1200 U/ml for GADA seemed to separate ‘far’ vaccinated participants into clusters with higher and lower GADA levels, and was used to calculate cytokine levels for the two subgroups. Interestingly, participants within the group with high GADA titres seemed to also have higher IL-13, IL-5, IL-17, TNF-α, IFN-γ, IL-10 and IL-1β levels compared with participants with lower GADA titres, although this data did not reach statistical significance (ESM Fig. 2).

### Change in fasting C-peptide and AUC among H1N1-vaccinated participants

**Fig. 4:**
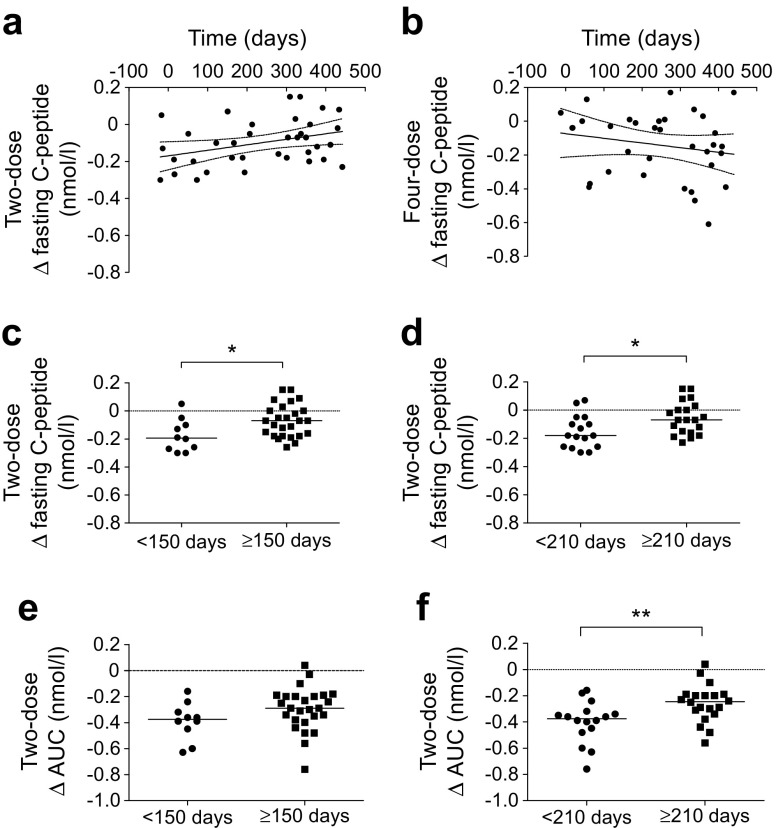
Change (Δ) in fasting C-peptide (baseline to 15 months) for each participant and its correlation with the relative time (days) between the first injection of GAD-alum and receipt of the H1N1 vaccine in the (**a**) two-dose and (**b**) four-dose groups. Significant correlation in the two-dose group was found between Δ fasting C-peptide and the relative time between vaccinations (*p* = 0.029, *r* = 0.31). (**c**–**f**) Δ fasting and stimulated (AUC) C-peptide in the two-dose group from baseline to 15 months in H1N1-vaccinated participants. Cut-offs of 150 and 210 days between H1N1 vaccination and GAD-alum treatment were used to define ‘close’ (<150 and <210 days, circles) and ‘far’ (≥150 and ≥210 days, squares) vaccinations. For (**c**–**d**), data are presented as median values. *p < 0.05 and **p < 0.01, ‘close’ vs ‘far’ H1N1 vaccination

We next assessed whether there was any association between the relative time between GAD-alum and H1N1 injections and the change in fasting and stimulated (AUC) C-peptide from baseline to 15 months. Intriguingly, a positive correlation was observed between the change in fasting C-peptide and the relative time between the two treatments in the two-dose group (*p* = 0.029, *r* = 0.31), but not in the four-dose group (*p* = 0.13, *r* = −0.19) (Fig. 4a, b). No association was found between the change in AUC and the relative time between treatments in either the two-dose or four-dose group (ESM Fig. 3). Despite this association in the two-dose group, we did not observe any difference in the loss of fasting C-peptide or AUC at 15 months between participants who received the H1N1 vaccine and those who did not (ESM Fig. 4). However, comparison of ‘close’ and ‘far’ vaccinated individuals, using both the 150 and 210 days cut-off period, showed that ‘close’ vaccinated individuals within the two-dose group had greater reduction in fasting and stimulated C-peptide at 15 months compared with ‘far’ vaccinated participants in the same group (Fig. 4c–f), although the reduction in stimulated C-peptide was non-significant using the 150 days cut-off value. No differences were observed between ‘close’ and ‘far’ vaccinated participants in the four-dose group (ESM Fig. 5).

## Discussion

In this study, we evaluated whether vaccination against the H1N1 virus with the Pandemrix vaccine could have influenced the specific immune response induced by GAD-alum treatment and, if so, whether an effect was also seen in residual insulin secretion. It was interesting to observe that GADA levels were associated with the relative time between GAD-alum and H1N1 injections in the two-dose group, and that GADA levels were higher in participants who received the GAD-alum and H1N1 injections more than 210 days apart, compared with those who received them closer together. Consistent with the GADA results, we also observed increased levels of several GAD_65_-induced cytokines in participants within the two-dose group who were given GAD-alum and H1N1 injections ‘far’ apart (≥210 days) compared with those who received them ‘close’ (<210 days). Intriguingly, participants within the two-dose group who received GAD-alum and the H1N1 vaccine ‘far’ apart had, in parallel to higher GADA and GAD_65_-induced cytokine levels, a significantly smaller decline in both fasting and stimulated C-peptide, as compared with those vaccinated ‘close’ to GAD-alum treatment. Together, our findings suggest that vaccination with Pandemrix within a certain time frame with respect to the first GAD-alum injection seems to interfere with both the specific immune response induced by GAD_65_ and C-peptide preservation.

It is difficult to explain why the association between GADA levels and the relative time between the GAD-alum and H1N1 injections, and the differences between ‘close’ and ‘far’ vaccinated individuals observed in the two-dose group were not evident for the four-dose group. We have previously shown that the small number of participants in the two-dose GAD-alum group who completed the 30 months’ visit of the Phase III GAD-alum trial had significantly less decline in both fasting and stimulated C-peptide, and a significantly larger proportion of these participants retained more than 25% of their initial C-peptide, while C-peptide in the placebo and four-dose groups continued to decline [19]. Results from the trial showed that a predominant secretion of GAD_65_-induced proinflammatory cytokines early after treatment was associated with low stimulated C-peptide at 30 months in participants from the two-dose group [15]. Interestingly, in the current study, we found that the Th2 cytokines, IL-5 and IL-13, contributed to approximately 80% of the cytokine profile of ‘far’ vaccinated participants in the two-dose group using the 150 days cut-off, suggesting a stronger Th2 deviation in the GAD_65_-specific immune response in ‘far’ vaccinated individuals.

Although the investigation of immune interference with combined vaccination and the assessment of immune responses to vaccinations are important parts of vaccine development, the possible interference of vaccines with autoantigen treatment in type 1 diabetes is a poorly explored field. According to our results, the reduced specific immune response to GAD_65_ in individuals with a shorter relative time between H1N1 and GAD-alum injections suggests that the powerful immunomodulatory effect of the adjuvant AS03 used in the Pandemrix vaccine might have affected the specific immune response induced by GAD-alum.

Detection of differences in the immune response when patients vaccinated against H1N1 were compared as ‘close’ and ‘far’ using relative periods longer than those accepted in the Phase III trials for other vaccinations raises the question as to whether the interference of other vaccines with autoantigen administration might last longer than previously believed.

Of course, immune interference between treatments is not always clinically important, but our observation of lower C-peptide levels in participants with a diminished GAD_65-_induced immune response is very interesting, and supports the hypothesis that Pandemrix vaccination in Sweden and Finland may have affected the outcome of the Phase III GAD-alum trial.

It has recently been suggested that the high immunogenicity of H1N1 vaccines formulated with AS03 seems to be due to the capacity of the adjuvant to both stimulate increased activation of naive B cells, reducing immune interference with previous vaccines, and increase the adaptation of pre-existing memory B cells, giving improved specificity to the H1N1 vaccine [20]. Whether the effect observed by us is due to the potency of this specific adjuvant used in Pandemrix, or to mechanisms that may be relevant for other adjuvants or simultaneous vaccinations, should be addressed in future studies. Different vaccines stimulate the immune system in different ways, with some providing a stronger and broader response than others, as determined by the nature and amount of antigen, the route of administration and the adjuvant used. Adjuvants impact the kinetics and magnitude of both T and B cell responses to a given antigen. For instance, it has recently been reported that five different adjuvants formulated with the same antigen induced different adaptive responses, with a lower reactogenicity and lower magnitude of T and B cell responses in the group where the antigen was alum-formulated [21]. Low immunogenicity of alum-formulated vaccines raises the question as to whether other vaccines should be avoided close to GAD-alum immunotherapy. It is important to note that, despite being an attractive therapeutic modality for years, immunotherapy with autoantigens in type 1 diabetes has not been successfully translated into clinical practice, supporting the importance of applying results and experiences from previous trials into new clinical studies and into the development of efficacious treatments.

There are some limitations to be considered in this study. Because WHO had declared a pandemic alert for the influenza A/(H1N1)pdm09 strain, very few participants enrolled in the Phase III GAD-alum study declined to receive the influenza vaccination at the time of the study. This resulted in a low number of H1N1 non-vaccinated participants, a factor limiting the statistical assessment between vaccinated and non-vaccinated individuals within each GAD-alum treatment arm. The same issue limited the analysis of the effect of the H1N1 vaccine given before or after the first injection of GAD-alum, since this stratification led to very few individuals in the group who received H1N1 before the first injection of GAD-alum.

It would also have been very interesting to be able to compare the immune responses between Swedish participants and those from other European countries but collection of PBMC for immunological analysis was only performed in the Swedish population during the Phase III trial. Further, it would also have been relevant to analyse the specific cytokine profile induced by H1N1 vaccination, but limited sample volume precluded stimulation of PBMC with H1N1.

In conclusion, overall our results support the idea that H1N1 vaccination might have had an impact on the outcome of the Phase III GAD-alum trial. This impact was mainly seen in participants from the two-dose GAD-alum treatment group, where immunological and clinical variables seemed to be affected by the relative time difference between H1N1 vaccination and GAD-alum treatment. Signs of possible vaccine interference were observed far beyond the time period during which concomitant vaccinations, excluding influenza, were disallowed in the Phase III GAD-alum trial. This should be taken into consideration in the design of future clinical trials of autoantigen treatment.

## Electronic supplementary material

ESM (PDF 257 kb)

## Abbreviations

GADA: Glutamic acid decarboxylase autoantibodies
GAD-alum: Glutamic acid decarboxylase formulated with aluminium hydroxide
H1N1Ab: H1N1-haemagglutinin antibody
PBMC: Peripheral blood mononuclear cell
Th: T helper
