# Supplementary material for: Effect of simultaneous vaccination with H1N1 and GAD-alum on GAD65-induced immune response
Source: Diabetologia. 2017 Mar 29;60(7):1276–83. doi: 10.1007/s00125-017-4263-x (PMC5487599; doi:10.1007/s00125-017-4263-x)
Supplement: Supplementary file 1 — (PDF 257 kb) [file 125_2017_4263_MOESM1_ESM.pdf]

**ESM Table 1. Levels of cytokine secretion upon in vitro PBMC stimulation with $GAD_{65}$ within H1N1 vaccinated patients in two dose and four dose groups.**

| | | Cytokine profile 15 months | | | | | | | |
|---|---|---|---|---|---|---|---|---|---|
| | | IL-13 | IL-5 | IL-17 | TNF | IFN | IL-10 | IL1b | IL-2 |
| Two dose | <150 days (*n*=8) | 139.94 | 59.15 | 0.00 | 19.15 | 72.56 | 0.66 | 0.00 | 7.82 |
| | ≥ 150 days (*n*=23) | 1512.47 | 316.85 | 20.56 | 68.11 | 333.40 | 4.54 | 4.45 | 0.15 |
| | <210 days (*n*=13) | 389.00 | 97.56 | 0.00 | 30.94 | 81.47 | 0.91 | 1.72 | 8.18 |
| | ≥210 days (*n*=18) | 1635.35 | 351.33 | 22.60 | 61.65 | 340.05 | 5.24 | 4.01 | -0.19 |
| Four dose | <150 days (*n*=7) | 1178.27 | 1116.37 | 18.96 | 92.82 | 366.82 | 13.48 | 11.22 | 0.00 |
| | ≥150 days (*n*=22) | 1059.19 | 710.93 | 13.70 | 51.77 | 253.72 | 4.58 | 4.77 | 0.00 |
| | <210 days (*n*=10) | 2207.20 | 1034.40 | 14.59 | 86.27 | 313.44 | 11.24 | 8.90 | -0.10 |
| | ≥210 days (*n*=19) | 841.75 | 665.90 | 17.17 | 57.64 | 247.38 | 4.24 | 4.49 | 0.00 |

Antigen-induced cytokine secretion are given after subtraction of spontaneous secretion. Median levels of IL-13, IL-5, IL-17, TNF, IFN-g, IL-10, IL-1b, IL-2 (pg/ml) were detected by Luminex. Subdivision was performed according to the relative time between influenza and GAD-alum injections using 150 day and 210 days cut-off.

**ESM Fig. 1**

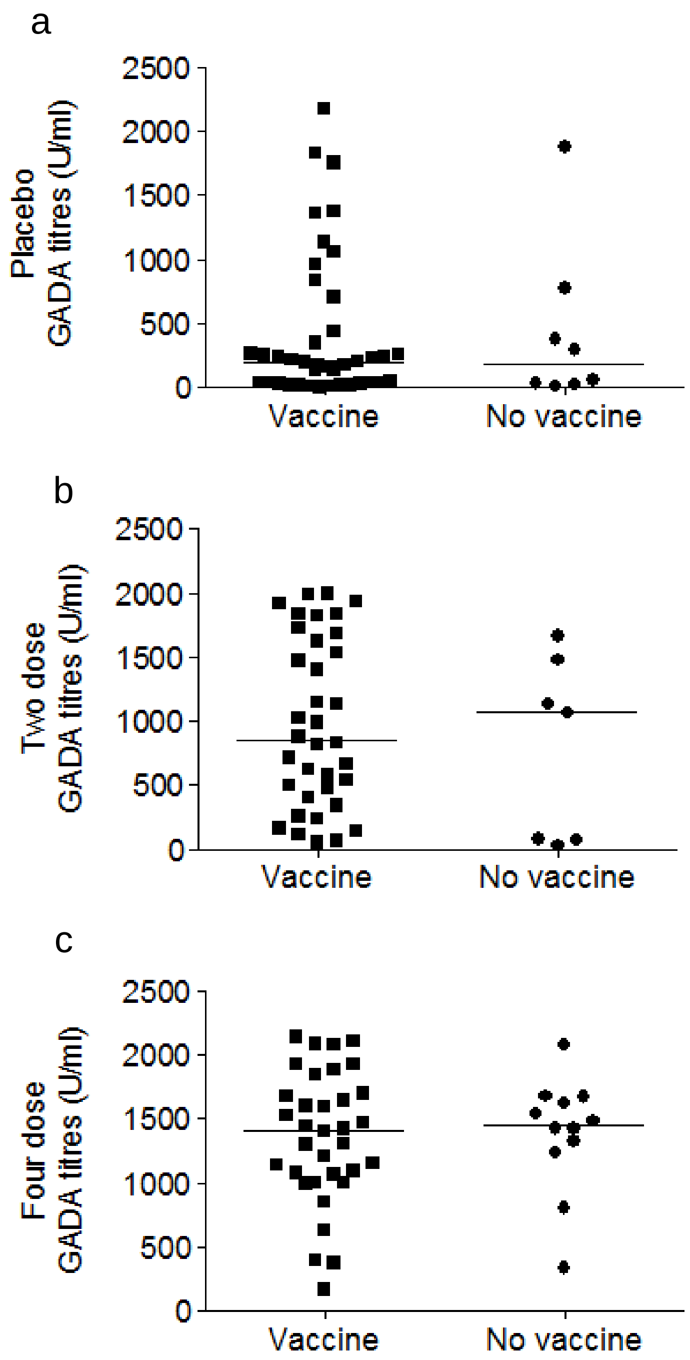


**GADA levels (a-c) at 15 months in patients who received GAD-alum (two dose and four dose) or placebo.** Patients within each arm were stratified according to whether they received H1N1 vaccination (black squares) or not (black circles). Median values are indicated by horizontal lines. Levels of GADA are expressed as U/ml. Differences between groups were calculated by Mann-Whitney U-test.

**ESM Fig. 2**

a

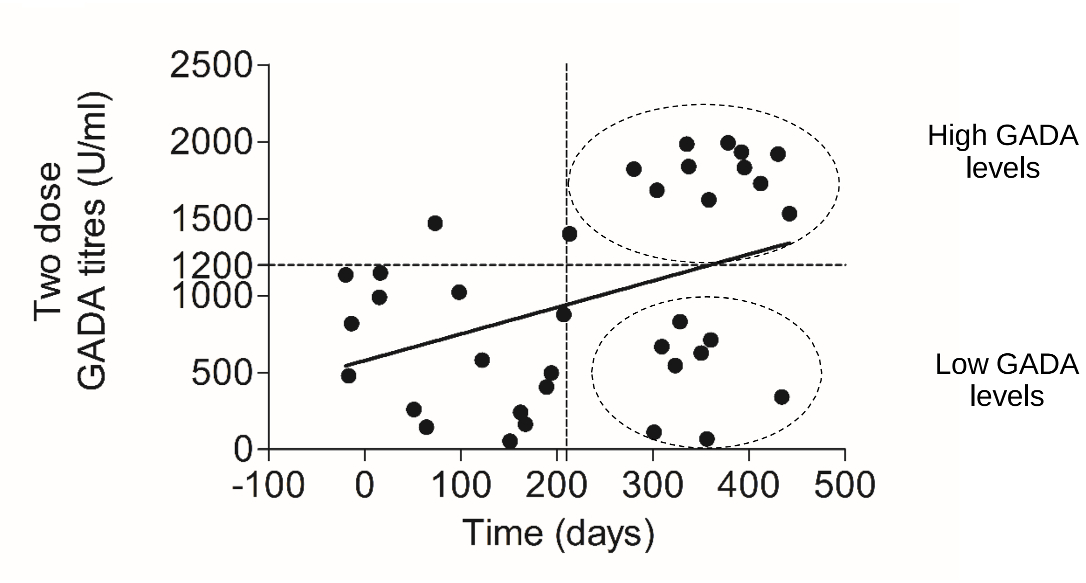


b

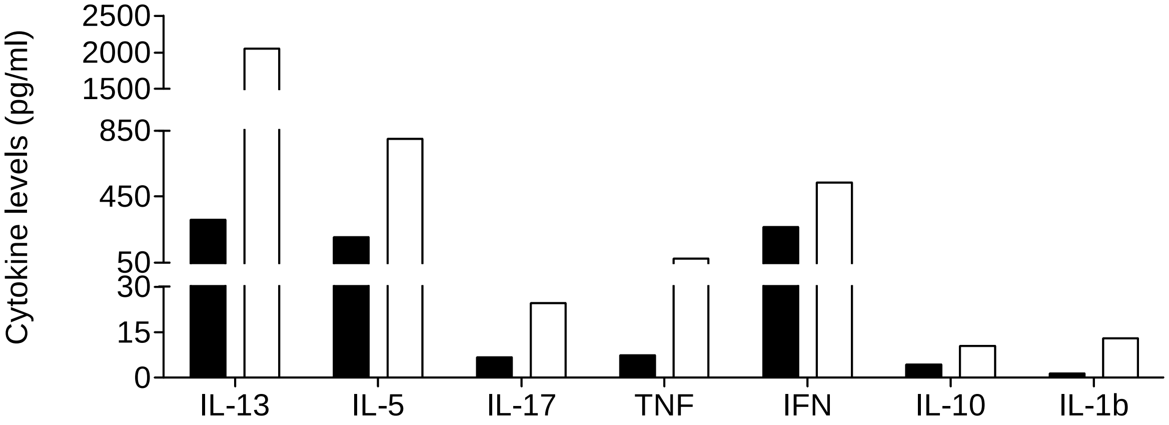


**GADA titers in the two dose group (a) scattered in relation to the relative time between GAD-alum and H1N1 vaccinations**. A cut-off of GADA at 1200 U/ml (horizontal discontinue line) separated the far vaccinated patients (210 days cut-off) in two differential clusters with “high” and “low” GADA . Cytokine profile (b) in patients with “high” GADA (white bars) seemed to also have higher cytokine secretion compared to “low” GADA (black bars) individuals, without being statistically significant.

**ESM Fig. 3**

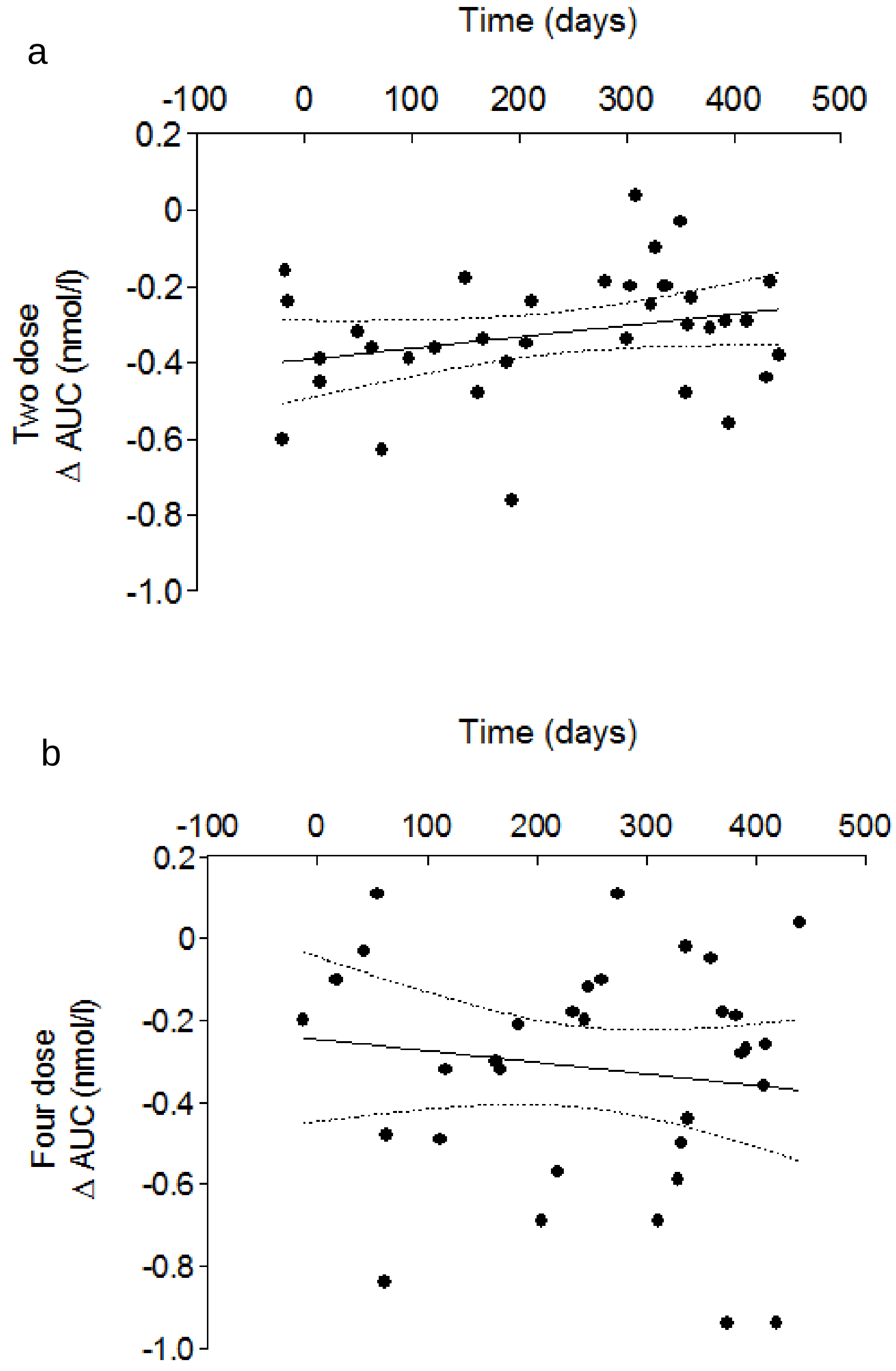


**Change (Δ) in stimulated C-peptide (AUC) from baseline to 15 months for each patient and its correlation with the relative time (days) between the first injection of GAD-alum and H1N1 vaccine in the two dose (a) and four dose (b) group.** AUC is expressed as nmol/l. Correlation were calculated using Spearman correlation analysis.

**ESM Fig. 4**

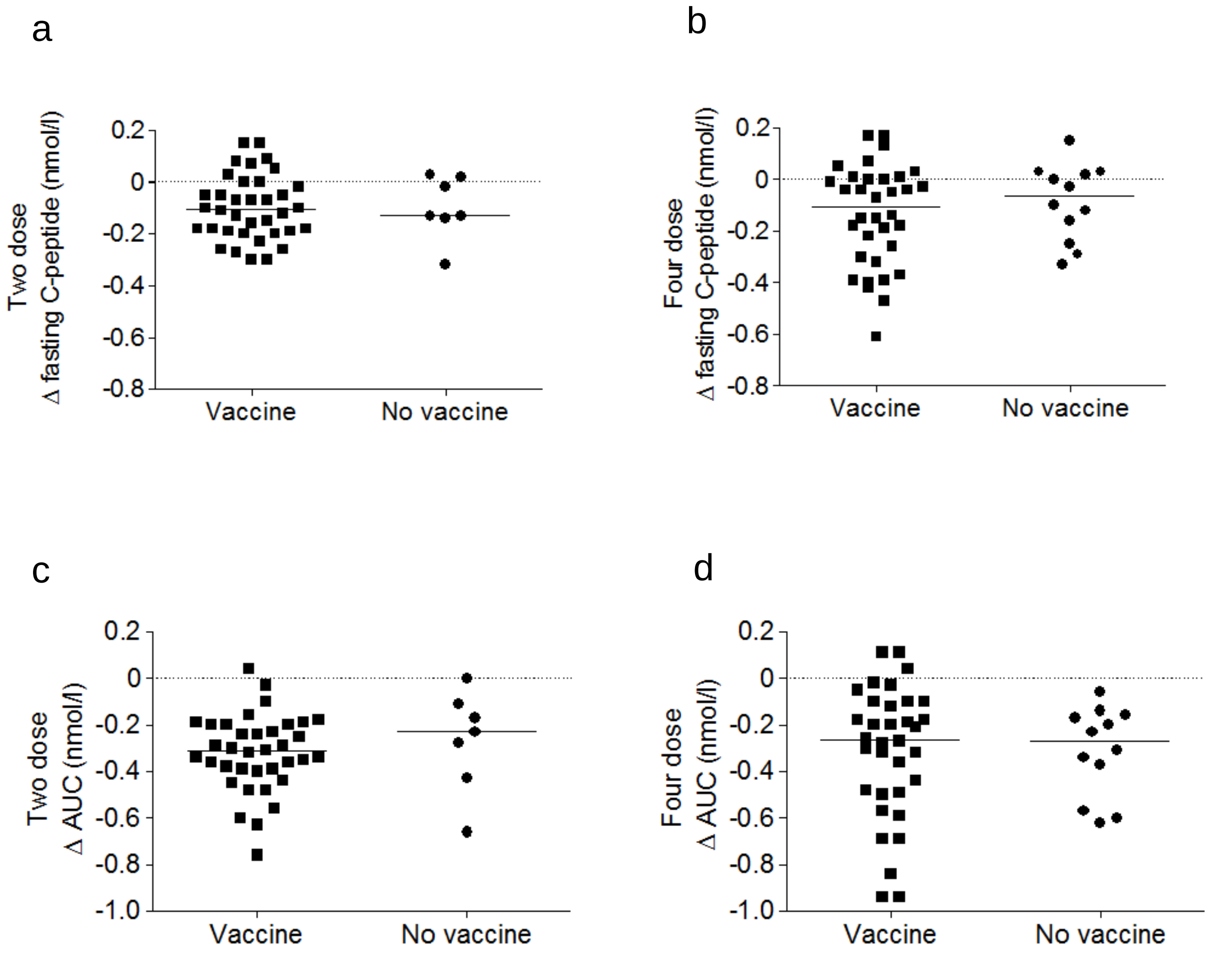


**Comparison of the change (Δ) in fasting and stimulated (AUC) C-peptide from baseline to 15 months in patients vaccinated or not against H1N1 among patients receiving two dose (a-c) or four dose (b-d) of GAD-alum.** Median values are indicated by horizontal lines. Discontinue line represents value 0 (no change). Differences between groups were calculated by Mann- Whitney U-test.

**ESM Fig. 5**

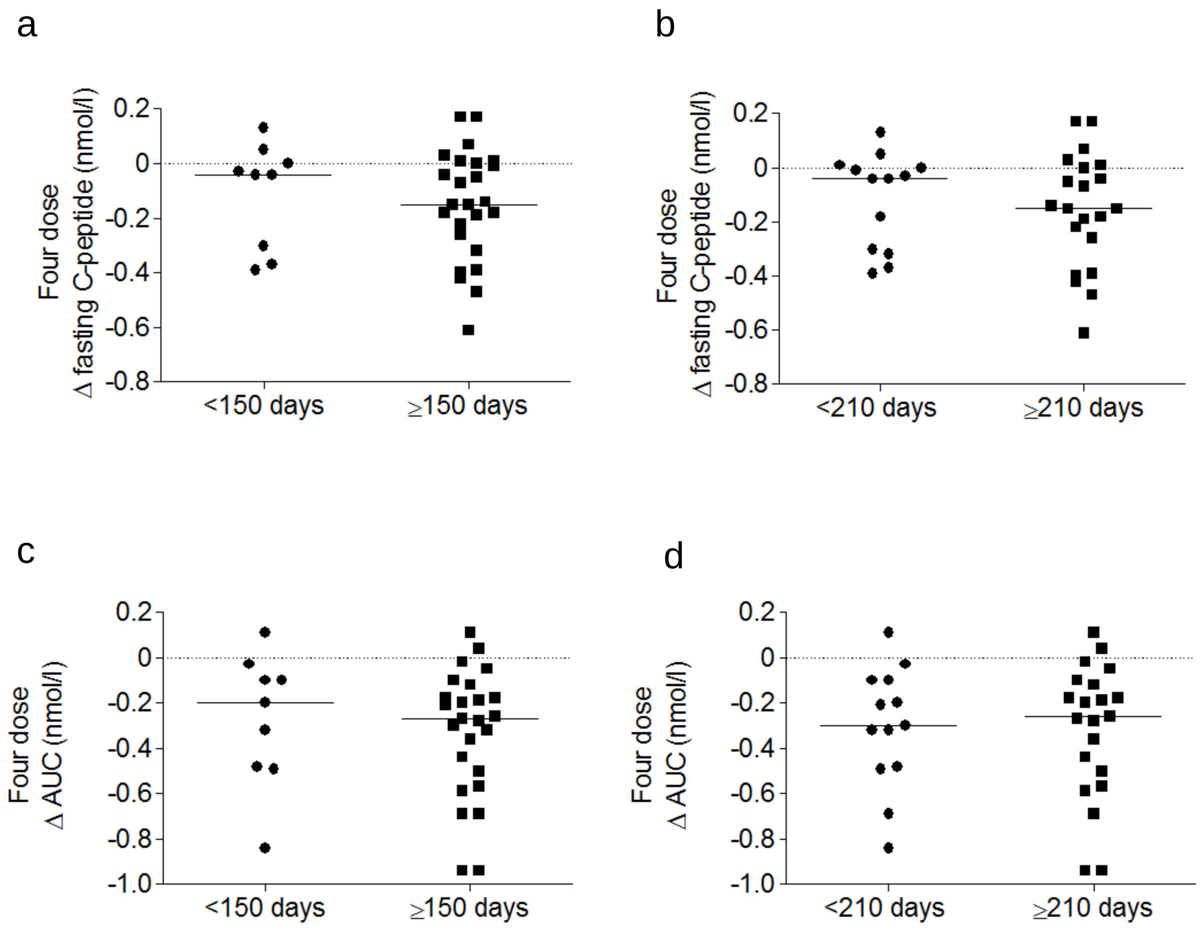


**Fasting C-peptide and AUC change (Δ) from baseline to 15 months according to the relative time between influenza and GAD-alum injections in patients receiving four dose of GAD-alum.** Patients were divided according to the relative time between influenza and GAD-alum injections using the two different cut-off of 150 days (**a-c**) and 210 days (**b-d**) to define close (<150 and <210 days respectively) and far (≥150 and ≥210 days respectively) vaccination. Median values are indicated by horizontal lines. Discontinue line represents value 0 (no change). Differences between groups were calculated by Mann-Whitney U-test.
